# Supplementary material for: Omalizumab Restores Response to Corticosteroids in Patients with Eosinophilic Chronic Rhinosinusitis and Severe Asthma
Source: Biomedicines. 2021 Jul 7;9(7):787. doi: 10.3390/biomedicines9070787 (PMC8301363; doi:10.3390/biomedicines9070787)
Supplement: Supplementary file 1 [file biomedicines-09-00787-s001.zip › Supplementary Materials_Oma_Biomedicines 2021 (final).pdf]

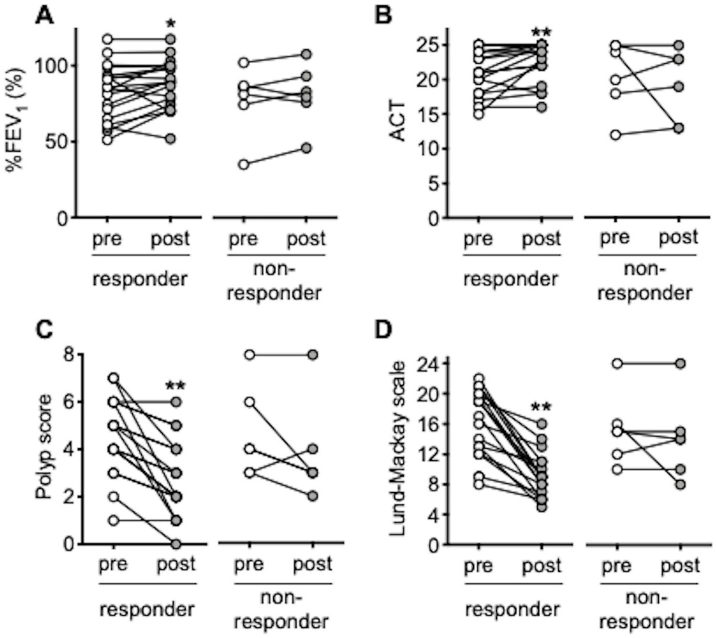

Figure S1. Effect of omalizumab on pulmonary function and sinusitis. FEV_1 (**A**), Asthma Control Test (ACT) (**B**), polyp score (**C**) and sinus CT score (**D**) were evaluated pre- and post-treatment with omalizumab. Individual values of the responder group (n = 19) and the non-responder group (n = 6) are shown; * $P < 0.05$, ** $P < 0.01$ (vs. pre-treatment).

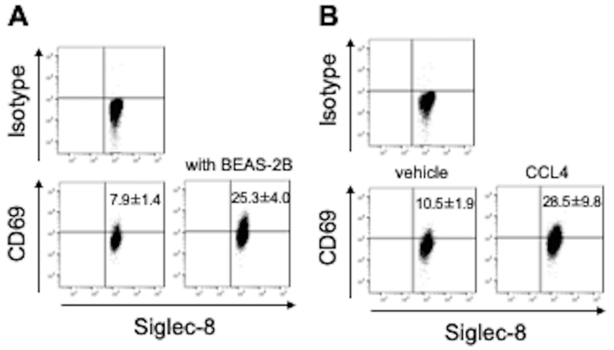

Figure S2. CD69 expression on human eosinophils. Purified peripheral blood eosinophils were incubated with BEAS-2B cells (A) or CCL4 (10 μg/mL) (B) overnight. CD69 expression on eosinophils was evaluated. Values in dot-plot panels (% of CD69+ Siglec-8+ double-positive cells) represent the mean ± SEM values of four experiments.

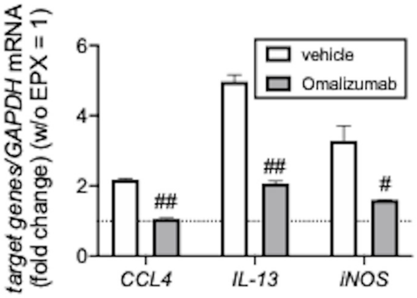

Figure S3. Effect of omalizumab on indicators of type 2 inflammation. BEAS-2B cells co-incubated with recombinant eosinophil peroxidase (EPX, 10μg/mL; Proteintech, Rosemont, IL, USA) for 72 h were treated with omalizumab (10 μg/mL) overnight. mRNA levels of CCL4, IL-13 and iNOS in BEAS-2B cells were evaluated. Values in panel represent the mean ± SEM values of three experiments; $^{\#}P$<0.05, $^{\#\#}P$<0.01 (vs. non-treatment control without EPX).

Table S1. Amplification primers (5′–3′)

gene	forward	reverse
PPP2CA	CGC CAT TAC AGA GAG CCG AG	GTA CTT CTG GCG GCT GTT GA
CCL4	CTC CTC ATG CTA GTA GCT GCC TTC	GGT GTA AGA AAA GCA GCA GGC GGT
IL-13	CTG ACA GCT GGC ATG TAC TGT G	TGT CTC GGA CAT GCA AGC TG
iNOS	ATC TCT GGT CAA GCT GGA TGC	GCC TTA TGG TGA AGT GTG TCT TG
GAPDH	TTC ACC ACC ATG GAG AAG GC	AGG AGG CAT TGC TGA TGA TCT
